# Diagnostic Challenges in Severe Electrolyte Imbalance in Early Infancy: A Case Report of Secondary Pseudohypoaldosteronism

**DOI:** 10.3390/pediatric18020049

**Published:** 2026-04-01

**Authors:** Stanimira Elkina, Irina Halvadzhiyan, Venetsiya Bozhanova

**Affiliations:** Department of Pediatrics, Medical University-Pleven, 5800 Pleven, Bulgaria

**Keywords:** secondary pseudohypoaldosteronism, electrolyte disturbances, congenital anomalies of the kidney and urinary tract, CAKUT, urinary tract infection

## Abstract

**Background:** Secondary pseudohypoaldosteronism (PHA) is a rare, transient condition caused by renal tubular resistance to aldosterone, most commonly associated with urinary tract infection (UTI) and/or congenital anomalies of the kidney and urinary tract (CAKUT). It mimics primary adrenal disorders, presenting with life-threatening electrolyte disturbances in early infancy. **Case Presentation:** We report a male infant admitted twice within the first four months of life with severe dehydration, hyponatremia, hyperkalemia, metabolic acidosis, and acute kidney injury (AKI). Urine cultures grew *Klebsiella pneumoniae* and later *Escherichia coli*. Imaging studies demonstrated obstructive CAKUT, including posterior urethral valves, bilateral megaureters, hydronephrosis, and bladder diverticulosis. Congenital adrenal hyperplasia was excluded. Further evaluation showed markedly elevated plasma renin and aldosterone levels, confirming secondary PHA. The patient was successfully treated with intravenous fluids, electrolyte correction, and antibiotic therapy. Subsequently, oral sodium chloride and bicarbonate supplementation were added. Stepwise surgical correction of the urinary tract anomalies was initiated. **Conclusions:** Secondary PHA should be considered in infants presenting with failure to thrive, dehydration, hyponatremia, and hyperkalemia, particularly in the presence of UTI or CAKUT. Early recognition and differentiation from primary adrenal disorders are essential to prevent life-threatening complications. Prompt correction of electrolyte imbalance and management of the underlying urinary tract pathology are crucial for favorable outcomes.

## 1. Introduction

Pseudohypoaldosteronism (PHA) is a clinical syndrome characterized by multiorgan or isolated renal tubular resistance to aldosterone, resulting in hyperkalemia, metabolic acidosis, and normal to elevated serum aldosterone levels [1]. PHA may be primary (hereditary) or secondary (acquired) [2,3].

Primary forms are divided into PHA type 1 (salt-wasting) and PHA type 2 (salt-retaining). PHA type 1 can be inherited in either an autosomal dominant or autosomal recessive manner. The autosomal dominant form is more common and is known as “renal form” because of a structural abnormality of the aldosterone receptors in the kidneys. The autosomal recessive form results from impaired epithelial sodium channel activity and is known as “systemic form” of PHA type 1, typically involving extra-renal manifestations in the respiratory, gastrointestinal, sweat gland, and salivary systems [3,4]. PHA type 2 (Gordon syndrome) is a predominantly autosomal dominant monogenic disorder caused by pathogenic variants in the *WNK1*, *WNK4*, *CUL3*, or *KLHL3* genes [5,6].

The secondary form of PHA is typically associated with severe urinary tract infections (UTIs), often combined with obstructive congenital anomalies of the kidneys and urinary tract (CAKUT). Secondary PHA predominantly occurs in the first year of life and clinically mimics PHA type 1. It is a rare disorder (ORPHA: 93164), with approximately 100 cases reported worldwide according to some data, predominantly in male infants [7]. In secondary PHA, tubular dysfunction may be caused by renal immaturity or tubular resistance to aldosterone through direct impairment of cellular responses or indirectly through excessive secretion of renal prostaglandins or cytokines. The electrolyte and acid-base changes are transient and typically resolve with healing of the underlying urinary tract disturbance. However, unrecognized PHA can lead to life-threatening hyperkalemia with severe dehydration and cardiopulmonary arrest [7,8,9].

We present a case of secondary pseudohypoaldosteronism due to a congenital anomaly of the kidneys and urinary tract in a male infant. Written informed consent from the child’s caregiver for presenting the case and patient’s images was obtained before publication.

## 2. Case Presentation

### 2.1. Perinatal History and Initial Presentation

The patient is a 4-year-old male who was delivered naturally from a fourth, unmonitored pregnancy to consanguineous parents. Detailed obstetric records were unavailable; however, the birth weight was 2700 g, and the birth length was 47 cm, both at the lower part of reference ranges but appropriate for the gestational age of approximately 38–40 weeks. He has two older sisters with mild developmental delay.

In the neonatal period, the infant experienced necrotizing enterocolitis, which was treated conservatively. During this severe illness episode, hyponatremia (serum sodium 119 mmol/L) was detected but resolved with supportive treatment at the local Neonatal department. At that time, secondary PHA was not suspected, and the hyponatremia was attributed to the acute gastrointestinal illness.

### 2.2. First Hospitalization (2 Months of Age)

Until the age of 4 months, the boy was admitted twice to the University Pediatrics Clinic–Pleven. In both admissions, he presented impaired condition with severe dehydration, hyponatremia, hyperkalemia, metabolic acidosis, and acute kidney injury (AKI) (Table 1).

At first hospitalization (2 months old), the infant presented with poor feeding, vomiting, and lethargy. Clinical examination revealed severe dehydration with sunken frontal fontanelle, dry mucous membranes, and decreased skin turgor. Laboratory investigations showed severe electrolyte abnormalities with no obvious gastrointestinal disorder present. (detailed in Table 1). Salt-wasting form of congenital adrenal hyperplasia (CAH) was excluded based on normal cortisol levels and normal 17-OH-progesterone (5.5 nmol/L; reference range < 30 nmol/L in infants aged 1–3 months). Secondary PHA was not considered. Urine culture grew *Klebsiella pneumoniae* (>100,000 colony-forming units/mL).

**Figure 1:**
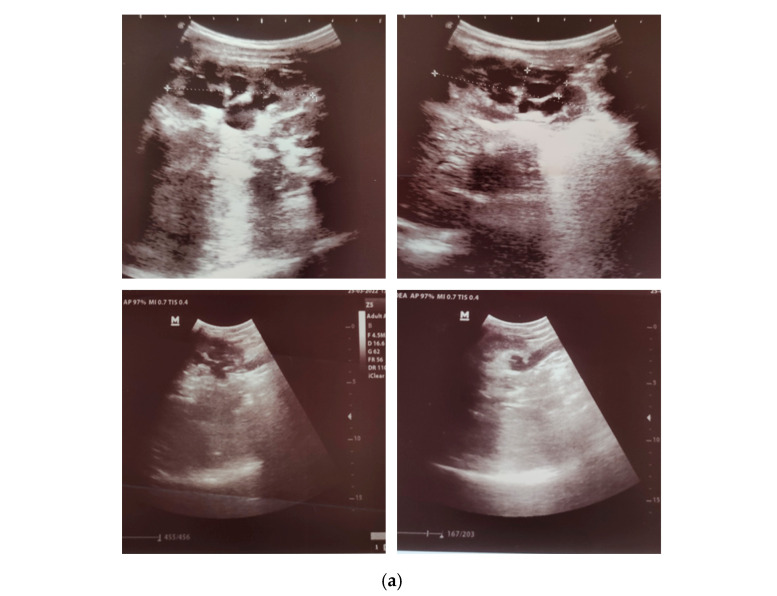

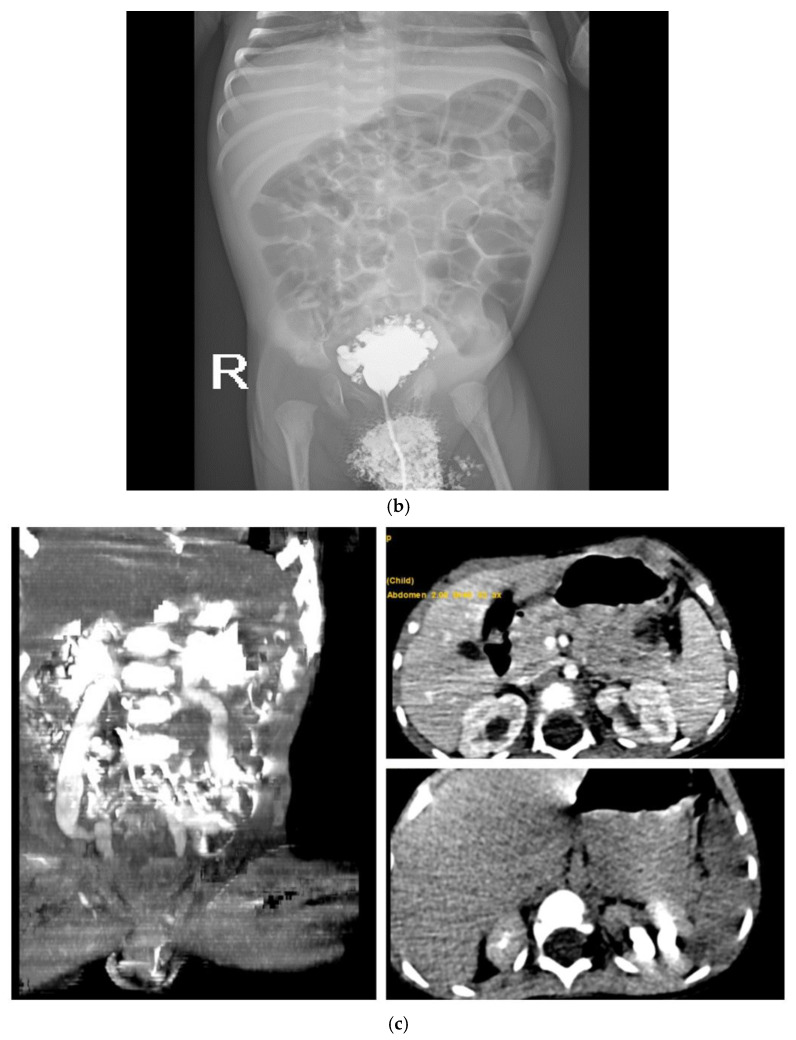
Imaging findings: (**a**) Bilateral Hydronephrosis with Hydroureters on Ultrasound; (**b**) voiding cystourethrography—bladder diverticulosis, no signs of vesicoureteral reflux; (**c**) CT scan of UTS—Bilateral Hydronephrosis with Hydrourethers.

Imaging investigations were performed to identify potential urinary tract pathology. Renal ultrasound revealed bilateral hydronephrosis with hydroureters (Figure 1a). Voiding cystourethrography demonstrated bladder diverticulosis without vesicoureteral reflux (Figure 1b). A computed tomography (CT) scan of the urinary tract system was performed to exclude complex congenital malformations not fully assessable by ultrasound. It confirmed bilateral hydronephrosis with hydroureters (Figure 1c). These findings led to the diagnosis of obstructive CAKUT with posterior urethral valves, bilateral megaureters, hydronephrosis, and bladder diverticulosis.

Treatment consisted of intravenous rehydration with normal saline, electrolyte correction (including calcium gluconate for hyperkalemia management), and broad-spectrum antibiotic therapy (Ceftriaxone). Feeding with a hydrolyzed formula due to concerns of cow’s milk allergy was initiated. The patient was discharged home in good general condition with appropriate oral intake and normalized serum electrolytes. Cystoscopy was not performed after this episode and diagnosis due to poor adherence and social factors on the part of the parents. The patient did, however, receive antibacterial prophylaxis for the prevention of UTI.

### 2.3. Second Hospitalization (4 Months of Age)

**Figure 2:**
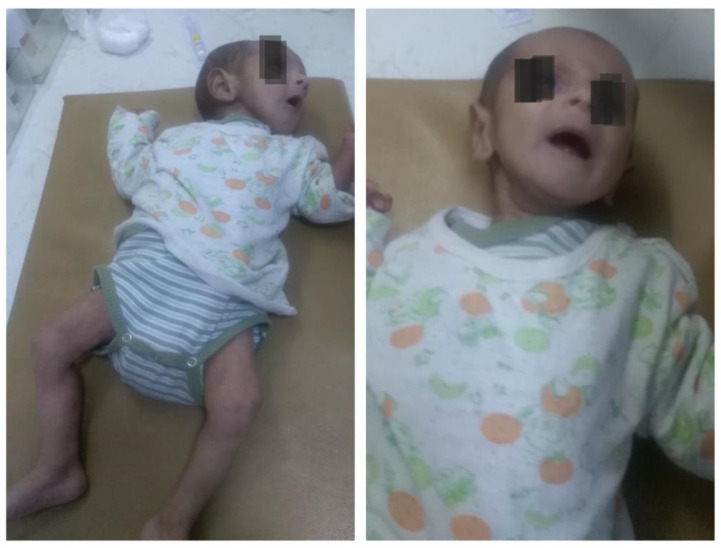
Clinical presentation at 4 months of age showed severe malnutrition and dehydration.

At the age of 4 months, the patient was readmitted in severe condition with a history of persistent feeding problems and recurrent vomiting over the preceding week. Physical examination revealed severe malnutrition approaching marasmus: weight 2800 g (<3rd percentile), length 56 cm (within normal range for age), sunken fontanelle, and markedly dry mucous membranes (Figure 2). The weight discrepancy reflects both acute dehydration and chronic failure to thrive. Before this acute presentation, the infant had demonstrated poor weight gain despite adequate caloric intake, consistent with ongoing electrolyte losses. Nutritional management included high-calorie hydrolyzed formula with additional sodium supplementation, though compliance at home was suboptimal.

Examination of the lungs, heart, and neurological system revealed no pathologies. Laboratory investigations again showed severe electrolyte disturbances and evidence of AKI. Urine culture grew *Escherichia coli* (>100,000 colony-forming units/mL) (Table 1).

Given the recurrent presentation with identical electrolyte abnormalities in the setting of UTI and known CAKUT, secondary pseudohypoaldosteronism was suspected. Hormonal evaluation was performed with the following results:

- Plasma renin: 467.4 mU/L (reference range 4.4–46.1 mU/L for infants aged 1–6 months);
- Aldosterone: >2770 pmol/L (reference range 139–2770 pmol/L for infants aged 1–12 months);
- Cortisol: 380 nmol/L (reference range: 138–690 nmol/L);
- 17-OH-progesterone: 12 nmol/L (reference range: <30 nmol/L).

These results confirmed markedly elevated plasma renin and aldosterone levels, consistent with aldosterone resistance and establishing the diagnosis of secondary PHA. It should be noted that these measurements were obtained after initial resuscitation and partial electrolyte correction but before complete normalization, which may have influenced absolute values. Ideally, renin and aldosterone should be measured during the acute phase before treatment; however, the severity of the clinical presentation necessitated immediate treatment interventions.

Treatment included intravenous rehydration, electrolyte correction (including hyperkaliemia), antibiotic therapy (Meropenem), and followed by initiation of oral sodium chloride (3–4 mEq/kg/day) and sodium bicarbonate (2–3 mEq/kg/day) supplementation in the later stage. The patient showed excellent laboratory response with normalization of electrolytes within 5 days. Following medical stabilization, stepwise operative correction of the CAKUT was initiated. Ongoing management includes regular urology follow-up and continued electrolyte supplementation.

## 3. Discussion

Secondary PHA is a rare condition of acquired renal tubular aldosterone resistance typically seen in infants with urinary tract infection (UTI) and/or congenital anomalies of the kidney and urinary tract (CAKUT). It presents with hyponatremia, hyperkalemia, metabolic acidosis, and elevated renin and aldosterone levels, and usually resolves with treatment of the underlying infection or obstruction. Rare cases have also been reported without overt urinary pathology [1,10,11].

Our patient is a male infant with secondary PHA and CAKUT who presented with severe dehydration, vomiting, and feeding problems, which is consistent with available literature. According to published data, most cases (approximately 90%) are diagnosed during the first six months of life, and more than 80% of affected children are males [7]. The male predominance is likely related to the higher incidence of obstructive uropathies, particularly posterior urethral valves, in male infants. According to Orphanet, fewer than 100 cases of secondary PHA have been reported worldwide; however, the number of published cases has been rising in recent years to 124 cases according to systemic review of Betti and all, 2024 [10], suggesting increased clinical awareness.

### 3.1. What Made This Case Challenging

Several factors contributed to diagnostic complexity in our case. First, the initial episode of hyponatremia during the neonatal period was attributed to necrotizing enterocolitis and did not trigger further endocrine investigation. Second, the non-specific clinical features—fatigue, vomiting, and poor feeding—are common to many pediatric conditions. Third, the patient’s consanguinity and family history of developmental delay raised concerns for genetic, metabolic, or endocrine disorders, requiring systematic exclusion of multiple differential diagnoses, including congenital adrenal hyperplasia CAH. Finally, the severity of malnutrition masked the underlying electrolyte disturbances, as clinical attention initially focused on nutritional rehabilitation.

The diagnosis became clear only after the second admission, when the pattern of recurrent electrolyte crisis in association with UTI and known CAKUT prompted specific testing for PHA. This case underscores the importance of maintaining a high index of suspicion for secondary PHA in infants with known urinary tract anomalies who present with unexplained electrolyte abnormalities.

### 3.2. Differential Diagnosis

Hyponatremia in combination with hyperkalemia and metabolic acidosis, which are typical for secondary PHA, are seen in multiple conditions. The primary differential diagnosis includes adrenal disorders, particularly CAH, which presents with salt wasting but is distinguished by low aldosterone levels and elevated 17-OH-progesterone. Although CAH is included in neonatal screening in Bulgaria and most high-income countries, some cases can be missed due to false-negative results, timing of sample collection, or administrative errors [12,13]. In our patient, CAH was definitively excluded by normal 17-OH-progesterone and cortisol levels in the setting of markedly elevated aldosterone.

Other differential diagnoses include isolated aldosterone deficiency and congenital adrenal hypoplasia, both less likely when aldosterone and cortisol levels are elevated, as seen in PHA [14]. Gastrointestinal losses of sodium and water during severe diarrhea, vomiting, or following extensive bowel resection can cause severe dehydration with impaired renal perfusion and clinical features resembling secondary PHA [15]. Our patient’s history of neonatal necrotizing enterocolitis raised this possibility; however, the persistent electrolyte abnormalities despite resolution of gastrointestinal symptoms, combined with elevated renin and aldosterone, pointed toward PHA rather than simple gastrointestinal losses. Patients with severe hyponatremia of unknown origin and hypotrophy should also be tested for cystic fibrosis. Though it usually presents with low potassium and alkalosis, performing a sweat test should be considered [8].

Primary (hereditary) PHA type 1 must also be excluded, as it clinically mimics secondary PHA. Primary PHA type 1 typically presents in the neonatal period with salt wasting and failure to thrive. The autosomal recessive (systemic) form results from mutations in genes encoding epithelial sodium channel subunits (*SCNN1A*, *SCNN1B*, *SCNN1G*) and affects multiple organ systems, not only the kidneys. Patients with systemic PHA type 1 exhibit more severe salt wasting and require lifelong sodium supplementation. In contrast, the autosomal dominant (renal) form is caused by mutations in the *NR3C2* gene encoding the mineralocorticoid receptor. Symptoms are limited to the kidney, severity diminishes with age, and salt supplementation is usually not required after 2 years of age. Also, in primary PHA-1, persistent renal resistance to aldosterone leads to ongoing salt-wasting, with both plasma renin activity and aldosterone markedly elevated, and electrolyte abnormalities that do not resolve without intervention. In contrast, secondary PHA shows transient elevations of renin and aldosterone during infection or urinary tract obstruction, with normalization and correction of electrolytes once the underlying cause is treated [3,12,16,17]. In our patient, the transient nature of the condition, resolution with treatment of underlying UTI and CAKUT, and lack of extra-renal manifestations supported the diagnosis of secondary rather than primary PHA. Long-term follow-up has confirmed complete resolution of electrolyte abnormalities following urological intervention, which would not be expected in primary forms.

### 3.3. Pathophysiology of Secondary PHA

The pathogenesis of secondary PHA remains incompletely understood. Aldosterone normally activates mineralocorticoid receptors on the principal cells of the cortical collecting duct, stimulating Na^+^/K^+^-ATPase and triggering potassium excretion and sodium reabsorption. In secondary PHA, this process is disrupted.

In our patient, the combination of obstructive CAKUT (posterior urethral valves with bilateral megaureters and hydronephrosis) and recurrent UTIs likely contributed to aldosterone resistance through multiple mechanisms. First, chronic obstruction could cause direct parenchymal injury to the renal tubules, impairing their responsiveness to aldosterone. Second, bacterial infection and associated inflammatory mediators—including endotoxins, prostaglandins, and transforming growth factor-β (TGF-β)—can decrease aldosterone receptor sensitivity Third, tubular immaturity in early infancy may exacerbate these effects, explaining why most reported cases occur in infants younger than 6 months of age [1,7,18,19].

The transient nature of the condition in our patient, with gradual improvement following antibiotic treatment and surgical correction of the obstruction, supports the acquired and reversible nature of aldosterone resistance in secondary PHA.

### 3.4. Management Principles of Secondary PHA

Early diagnosis and adequate treatment of secondary PHA are essential for favorable outcomes. Treatment focuses on rapid correction of life-threatening fluid and electrolyte imbalances and management of the underlying cause [10]. In the acute phase, intravenous fluid resuscitation, correction of hyperkalemia, and treatment of infection are priorities. Following stabilization, oral sodium chloride and sodium bicarbonate supplementation are typically required for several months. Definitive management involves the correction of underlying urological abnormalities. Most patients can be gradually weaned from electrolyte supplementation as renal tubular function matures and underlying pathology is corrected.

## 4. Clinical Learning Points

- Secondary PHA should be suspected in infants presenting with hyponatremia, hyperkalemia, metabolic acidosis, and dehydration, especially in the presence of UTI or CAKUT.
- Elevated plasma renin and aldosterone levels in the setting of hyponatremia and hyperkalemia differentiate secondary PHA from congenital adrenal hyperplasia and other adrenal disorders. Obtain these measurements before initiating treatment when clinically feasible.
- Infants with known CAKUT who develop unexplained electrolyte abnormalities during any systemic illness warrant evaluation for secondary PHA, even if UTI is not initially apparent.
- Early recognition and rapid correction of electrolyte imbalances are critical to prevent life-threatening cardiac arrhythmias and neurological complications.
- Treatment of the underlying urinary tract pathology, including prompt antibiotic therapy for UTI and surgical correction of anatomical abnormalities, leads to resolution of electrolyte disturbances in secondary PHA. Temporary electrolyte supplementation is typically required for several months because the renal tubular cells take time to fully recover from the insult that caused temporary aldosterone resistance.
- Long-term follow-up is necessary to monitor growth, renal function, and successful weaning from electrolyte supplementation as tubular function matures.

## 5. Conclusions

Although secondary PHA is a rare clinical condition, it should be considered in infants with failure to thrive, salt wasting, and hyperkalemia, particularly in the setting of UTI or CAKUT. Early screening for urinary tract anomalies should be performed in such cases to enable appropriate treatment and prevent life-threatening complications. Recognition of this condition requires a high index of clinical suspicion, as presenting features are non-specific and may be attributed to other common pediatric conditions. The transient and reversible nature of secondary PHA, with resolution following treatment of underlying urological pathology, distinguishes it from primary forms and emphasizes the importance of prompt diagnosis and comprehensive management.

## Figures and Tables

**Table 1:** Laboratory tests at baseline and on follow-ups.

Parameter (Reference Range)	WBC ×10^12^/L 6.0–17.5	Hb g/L 95–130	Plt ×10^9^/L 150–450	pH 7.35–7.45	HCO_3_^−^ mmol/L 22–26	BE mmol/L −2 to +2	Urea mmol/L 1.8–6.4	Cr µmol/L 18–35	UA µmol/L 143–340	Na mmol/L 135–145	K mmol/L 3.5–5.5	Cl mmol/L 98–107
1st Admission (2 months)	24.3	108	519	7.29	24.1	−1.9	6.0	102	296	132	6.3	100
2nd Admission (4 months)	32.2	96	1032	7.18	9.0	−18.5	38.3	127	695	123	8.4	80
Follow-up (4-year old)	10.2	119	370	7.38	21.3	0.2	6.2	30	210	128	5.2	101

Abbreviations: WBC, white blood cells; Hb, hemoglobin; Plt, platelets; HCO_3_^−^, bicarbonate; BE, base excess; Cr, creatinine; UA, uric acid; Na, sodium; K, potassium; Cl, chloride.

## Data Availability

The original contributions presented in this study are included in the article. Further inquiries can be directed to the corresponding author.

## Abbreviations

The following abbreviations are used in this manuscript:

PHA	Pseudohypoaldosteronism
AKI	Acute kidney injury
UTI	Urinary tract infection
CAKUT	Congenital anomalies of the kidney and urinary tract
CAH	Congenital adrenal hyperplasia
